# Extensive clinical experience: a simple guide to basal insulin adjustments for long-distance travel

**DOI:** 10.1186/2251-6581-12-59

**Published:** 2013-12-20

**Authors:** Jordan E Pinsker, Erik Becker, C Becket Mahnke, Michael Ching, Noelle S Larson, Daniel Roy

**Affiliations:** 1 Department of Pediatrics, Division of Endocrinology, Tripler Army Medical Center, 1 Jarrett White Road, Honolulu, HI 96859 USA; 2 Department of Pediatrics, Division of Cardiology, Tripler Army Medical Center, Honolulu, HI 96859 USA; 3 Department of Pediatrics, Developmental Pediatrics, Tripler Army Medical Center, Honolulu, HI 96859 USA

**Keywords:** Diabetes, Hypoglycemia, Insulin, Travel

## Abstract

Long-distance travel across multiple time zones presents unique challenges for patients taking insulin, requiring adjustments in both timing and dosage of basal insulin when several times zones (≥5) are traversed. Travel across the International Date Line adds to the confusion, as existing resources and dosing calculators often do not account for the date change. We review recommendations from available guidelines and dosage calculators used for long-distance travel basal insulin adjustments and then present our patient handouts which allow for a safe, specific, single dose adjustment for eastward and westward travel. The included handouts are easy to use and can be freely reproduced for use in diabetes clinics.

**Electronic supplementary material:**

The online version of this article (doi:10.1186/2251-6581-12-59) contains supplementary material, which is available to authorized users.

## Background

Patients with diabetes who take insulin face many unique challenges related to travel to include dehydration and hypoglycemia. Dehydration, a well documented risk associated with long-distance air travel, is accentuated in patients with diabetes; and extreme dehydration without proper insulin adjustment has been reported to trigger DKA [1]. Air travel also increases the risk of hypoglycemia, requiring adjustment of the dosage and timing of insulin administration [2].

Only a handful of resources offer travel guidance to patients who take insulin. These include publications targeting physicians, generalized information for patients and online dosage calculators. While they are all well written and offer good advice, many limit their scope to storage and transportation of supplies, immunizations and diet advice making them too generalized to answer specific insulin dosing questions [3–6]. Older publications generally review adjusting intermediate acting insulin (Lente, NPH), often used in combination with regular insulin, and are therefore less helpful for patients taking multiple daily injection (basal-bolus) regimens [7, 8]. Other guidelines, while extremely detailed and well done, are lengthy and contain complex accompanying figures which can potentially be overwhelming for patients and providers looking for a simple guide to adjusting insulin [9, 10]. Few publications explicitly address the issue of crossing the International Date Line (IDL) with sufficient detail to create a patient-friendly insulin adjustment plan.

Electronic dosage calculators, while helpful, require the patient to enter a significant amount of information online [11]. We are not aware of any online calculator that consistently accounts for crossing the IDL.

Our diabetes clinic, in a tertiary care medical center located in Honolulu, Hawaii is only 2 time zones away from the IDL and provides care to children and adult patients from across the western Pacific. Given our remote location, patients presenting for care frequently travel across multiple time zones east or west and routinely ask how to adjust their insulin when traveling back and forth across the IDL. In response to this need, we have developed detailed yet user-friendly patient handouts allowing physicians to offer patient-specific basal insulin dosing adjustment recommendations for long distance travel using only a single dose change [see Additional file 1].

In this review we discuss general rules for adjusting insulin during travel for patients on multiple daily injection (basal-bolus) regimens. We also include our patient handouts for eastward and westward travel that incorporate a single insulin dose adjustment for basal insulin, and review examples for multiple travel scenarios. As there are only a limited number of publications that provide advice for adjusting insulin for travel, and little scientific evidence to support their recommendations, we must emphasize that the advice given in this review is expert opinion based on our extensive clinical experience and knowledge of insulin pharmacokinetics, and is not evidence based.

### General rules for travel while taking insulin

Avoidance of hypoglycemia is the well-accepted primary goal during air travel. Hypoglycemia reportedly occurs in up to 10% of patients taking insulin while traveling abroad, either during travel or in the first 24 hours after arrival [12]. Many factors contribute to this increased risk, including changes in dietary patterns and difficulties in adjusting insulin dose and timing appropriately [2]. To reduce this risk of hypoglycemia recommendations have included removal and replacement of syringe plungers to allow pressure equalization prior to use for in-flight injections, and reduction in the amount of air normally injected into the insulin vials [13]. Changes in air pressure also affect insulin pumps with a reduction in air pressure occurring during ascent potentially resulting in unintended insulin delivery, yet no specific practice guideline currently recommends changes to insulin pump settings for travel [14].

### Adjustments to insulin dosage

Although many physicians appear reluctant to offer detailed advice on adjusting insulin doses for travel [15], the goal is actually quite straightforward; design an insulin regimen that is very simple and avoids hypoglycemia, even if this leads to a short period of suboptimal glycemic control (hyperglycemia). In general it is best to avoid complicated advice, such as to give frequent rapid acting insulin doses until it is time for basal insulin at the new location. In our experience complicated plans generally confuse patients and are therefore not well followed. Instead, adjusting basal insulin with a single dose change is the simplest method.

Short duration trips are typically easy. When traveling across fewer than 5 time zones or for trips less than 3 days duration, we recommend patients keep their watches set at their home local time, continue their basal insulin at the usual times and administer bolus doses before meals. Similarly, patients on insulin pumps taking only rapid acting insulin continue with their usual regimen regardless of duration of travel, simply adjusting the time on their insulin pump to local time on arrival so their basal rates adjust accordingly.

Traveling across 5 or more time zones and staying in a different time zone longer than 3 days disrupts meal times and medication dosages/times such that patients need a detailed plan to ensure a seamless transition [10, 16]. As noted above, similar to short duration travel, patients on insulin pumps taking only rapid acting insulin continue with their usual regimen, adjusting the time on their insulin pump to local time on arrival so their basal rates adjust accordingly. Rapid acting bolus doses via insulin pump or subcutaneous injection are still given before meals. However for patients taking long acting basal insulin, travel requires a 4% adjustment to the insulin dose for each time zone traversed (1 hour is 4% of the 24 hour day); with westward travel resulting in missed doses and eastward travel resulting in excess doses. Traveling across 5 time zones or more thereby necessitates a dose adjustment to prevent a clinically significant change in blood glucose.

In the following section, we offer 4 examples of long distance travel scenarios for patients on once or twice daily basal insulin regimens, demonstrate how to fill out our patient handouts and provide detailed yet easily understood patient instructions for basal insulin dosage adjustment.

### Example 1. Westward travel from New York to Honolulu

#### Scenario #1 – once daily basal insulin adjustment

Patient A with type 1 diabetes (T1D) is traveling west from New York to Honolulu. He currently takes glargine 20 units at 8 PM every night and rapid acting insulin before meals as part of his basal-bolus regimen. The flight departs at 10 AM Eastern Standard Time (EST). Total travel time is 11 hours.

**Figure 1:**
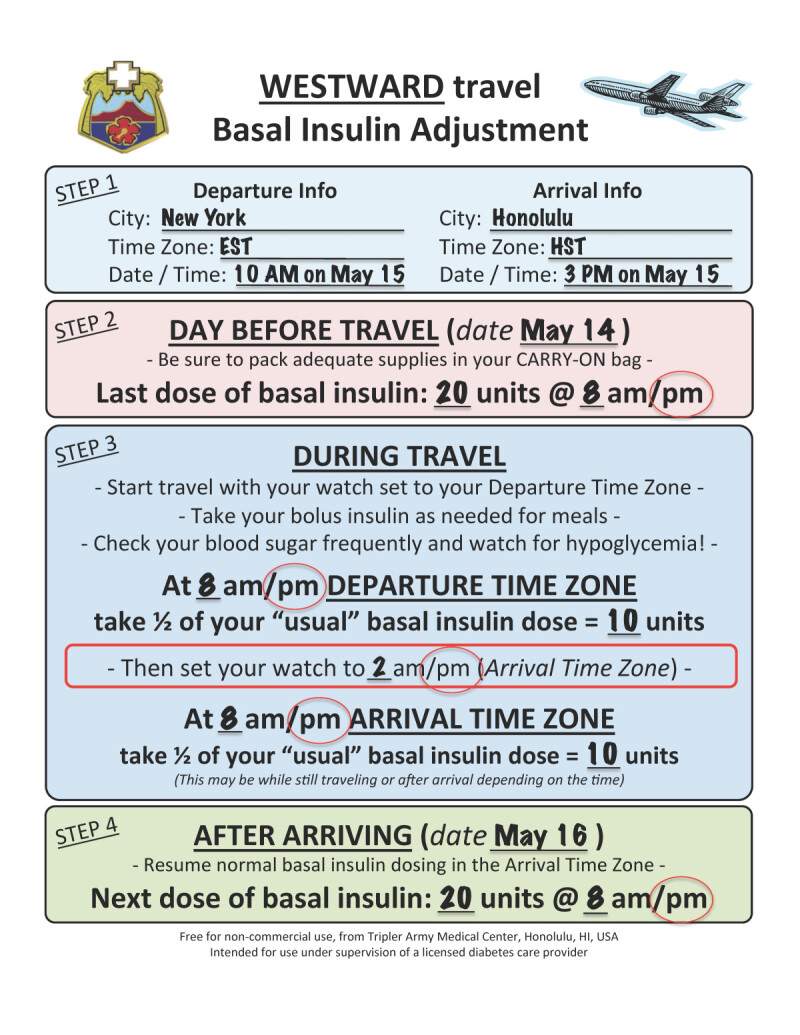
**Calculation for insulin dose change.** This example shows the basal insulin dose change for example 1, scenario 1, New York to Honolulu.

Using the Westward Travel Handout (Figure 1), information on the starting time at departure is recorded along with the last time basal insulin was given; in this case glargine 20 units at 8 PM the prior evening. The notes in the middle of the handout are there to remind patients to 1) continue to use rapid acting insulin during their flight as they would normally do, adjusting for the amount of food eaten; and 2) remind them that if their basal insulin is due during flight, *they should only take half of it* **(half the normal dose)**.

His watch should still be on departure time (EST) when due his next basal insulin dose, he takes half the normal dose (10 units glargine) at 8 PM EST. **AFTER** the basal insulin is given, he changes his watch to the destination time. Upon landing in Honolulu, the day is now ‘longer’ despite 11 hours having passed as it is only 3 PM Hawaii-Aleutian Standard Time (HST). As only half the basal insulin was given earlier in the day, he can then give the other half (10 units glargine) at 8 PM HST, whether this is on the plane or after the plane has landed. In this case the second half of the dose is given after landing in Hawaii. The next night, he resumes his normal insulin dosing at the new location.

By giving half the dose at 2 different times, the glargine dose is extended out to cover the longer day. Although the patient receives less than optimal insulin coverage at either end of the dosing schedule, the primary goal of preventing hypoglycemia is achieved while still giving insulin in a simple manner.

Patients may ask an important question, “How do I know what the destination time is to switch my watch to?” We recommend using a smartphone to do this by adding the destination city to the standard worldclock app. The handout also includes a space to write this out in advance for the patient. Alternately most flight attendants would be happy to assist, but we recommend the entire form be completed before the patient starts his travels. Use of a time zone map (http://www.worldtimezone.com/wtz-pacific24.php) can help to determine how many time zones are traversed.

#### Scenario #2 – twice daily basal insulin adjustment

Patient B is traveling west from New York to Honolulu. Instead of once daily glargine, the patient takes detemir 10 units twice daily at 8 AM and 8 PM, and rapid acting insulin before meals as part of the basal-bolus regimen. Although the patient takes twice daily basal insulin, the same handout used in the previous example works well and can be used again. The same flight departs at 10 AM EST. Total travel time is 11 hours.

This scenario is very similar to the previous example as the patient takes the total dose of basal insulin the morning before departure. Because detemir does not last quite as long as glargine, a dose becomes due during the flight; half the total dose (5 units detemir) should be given at that time (8 PM EST). Upon arrival at Honolulu, the patient adjusts his watch to HST and the other half of the basal dose (5 units detemir) is given at 8 PM HST. The normal dosing regimen resumes starting at 8 AM again.

### Example 2. Westward travel from Los Angeles to Seoul, South Korea (across the IDL)

#### Scenario #1 – once daily basal insulin adjustment

The same formula works well when traveling across the IDL. Patient A is traveling west from Los Angeles to Seoul, South Korea. He currently takes glargine 20 units at 8 PM every night and rapid acting insulin before meals as part of his basal-bolus regimen. The flight departs at 11:00 AM Pacific Standard Time (PST) on February 1st from Los Angeles and because the flight crosses the IDL, it arrives the next day (February 2nd) at 9:00 PM Korean Standard Time (KST) in South Korea. Although the trip is very long (almost 18 hours), crosses the IDL, and has a 3-hour layover in Tokyo, using our handout overcomes these complexities. We can use our formula to devise a simple basal insulin dose adjustment.

**Figure 2:**
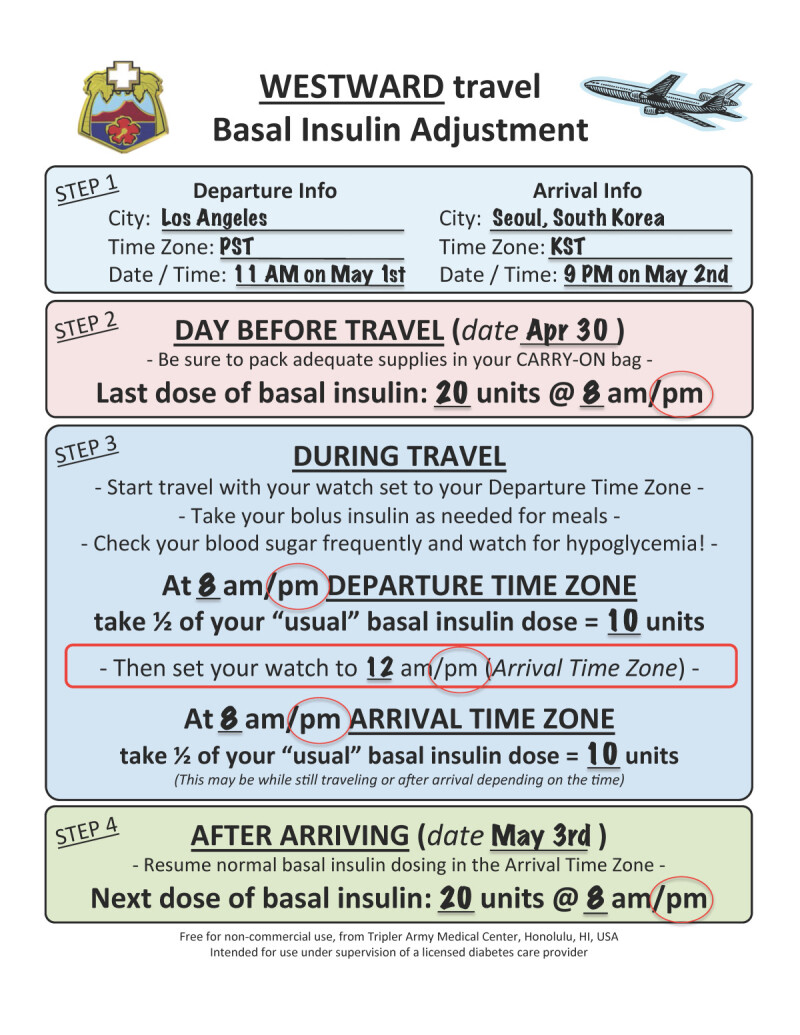
**Calculation for insulin dose change.** This example shows the basal insulin dose change for example 2, scenario 1, Los Angeles to Seoul, South Korea.

Using the Westward Travel Handout (Figure 2), information on the starting time at departure is written down as well as the last time basal insulin was given. In this case, glargine 20 units at 8 PM the prior evening. The patient’s watch should still be on departure time (PST) when 9 hours through the trip, his basal insulin becomes due. He gives half the normal dose (10 units glargine), then **AFTER** giving the basal insulin, he changes his watch to KST. In 8 more hours, it will be 8 PM KST. He still will not have arrived at the destination, but it does not matter. Because only half the basal insulin was given earlier in the day, he can then give the other half at 8 PM KST. He then resumes his normal insulin on at the new location, giving a full 20 units of glargine at 8 PM the next night on KST.

### Example 3. Eastward travel from Honolulu to New York

#### Scenario #1 – once daily basal insulin adjustment

Now let’s plan eastward travel for the same patient. When traveling east, it is important to recognize that the day gets shorter, so the basal insulin dose given during travel must be adjusted one time using the formula:

$$\text{Travel}\;\text{Dose}=\text{Normal}\;\text{Dose}\times \left(0.9-\frac{\#\text{of}\;\mathrm{Time}\;\mathrm{Zones}\;\mathrm{Crossed}}{\text{Hours}\;\text{Between}\;\text{Basal}\;\text{Insulin}\;\text{Doses}}\right)$$

This formula is adapted and modified from a previous reference [9]. The formula calculates the amount of insulin needed to bridge the gap between the departure dose (day before travel) and the dose given after arrival, accounting for the shorter day. The formula automatically reduces the amount of insulin given by 10-20%, depending on the length of travel, with a larger dose reduction calculated for longer flights, as longer flights are more likely to adversely affect diurnal rhythm and eating patterns, potentially increasing the risk of hypoglycemia. To see how it works, let us consider Patient A with T1D traveling home from Honolulu to New York. He currently takes glargine 20 units at 8 PM every night and rapid acting insulin before meals as part of his basal-bolus regimen. The flight departs at 3 PM HST and arrives 7 AM EST. Total flight time is 10 hours.

**Figure 3:**
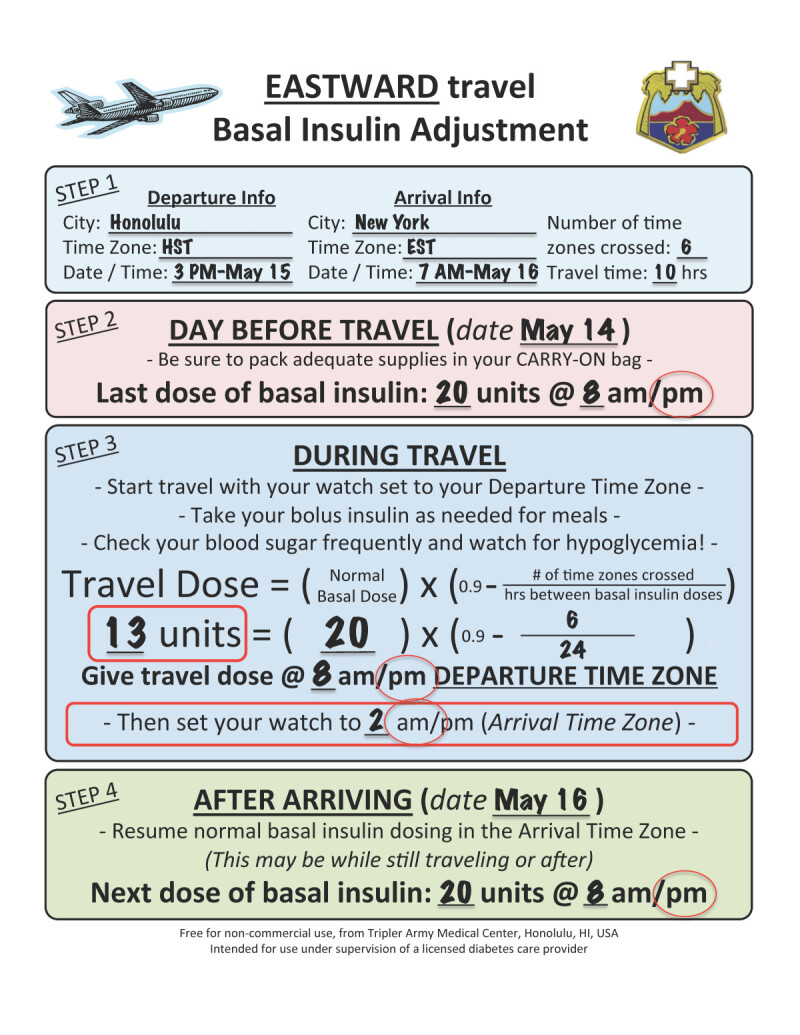
**Calculation for insulin dose change.** This example shows the basal insulin dose change for example 3, scenario 1, Honolulu to New York.

**Figure 4:**
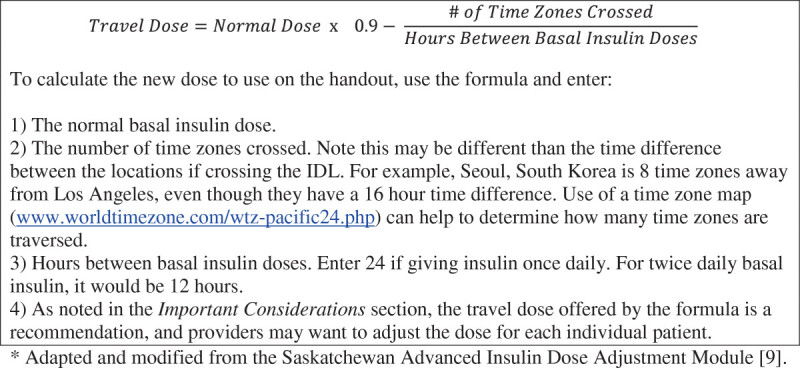
**Formula for eastward travel.** When traveling east, the day gets shorter. A reduced basal insulin dose is given once to account for the fact that the next dose will be due sooner than usual. The formula automatically reduces the amount of insulin given by 10-20%, depending on the length of travel, with a larger dose reduction calculated for longer flights, as longer flights are more likely to adversely affect diurnal rhythm and eating patterns, potentially increasing the risk of hypoglycemia.

Using the Eastward Travel Handout (Figure 3), we see that only a single dosage reduction is needed. After the reduced travel dose is given, the patient resumes their normal dosing. A detailed explanation of what information to put into the formula is discussed in Figure 4.

Information on the local time at departure is written down on the handout as well as the last time basal insulin was given before departure, glargine 20 units at 8 PM the prior evening. At 8 PM HST only 13 units of glargine are given per the formula on the handout. **AFTER** the basal insulin is given, the patient changes his watch to EST. The next full dose of glargine is due 8 PM EST after they arrive, which turns out to be 18 hours after the last dose. This reduced time between doses is why the patient took just under ¾ of his usual glargine dose on the flight.

#### Scenario #2 – twice daily basal insulin adjustment

Patient B is traveling home from Honolulu to New York. He currently takes detemir 10 units twice daily, at 8 AM and 8 PM and rapid acting insulin before meals as part of his basal-bolus regimen. The flight departs at 3:00 PM HST and arrives 7 AM EST. Total flight time is 10 hours. Although the patient takes twice daily basal insulin, the same handout used in the previous example works well and can be used again.

Using the Eastward Travel Handout, information on the local time at departure is written down as well as the last time basal insulin was given before departure, detemir 10 units at 8 AM that morning. The first dose of basal insulin due while traveling is at 8 PM Honolulu time. Per the formula on the handout only 4 units of detemir are given. **AFTER** the basal insulin is given, he changes his watch to EST. The next full dose of detemir is due 8 AM EST after he arrives, which turns out to be 6 hours after the last dose. This reduced time between doses is why the patient took just under ½ of his usual detemir dose on the flight, a dose that normally lasts 12 hours.

### Example 4. Eastward travel from Seoul, South Korea to Los Angeles (across the IDL)

#### Scenario #1 – once daily basal insulin adjustment

Patient A is traveling from Seoul, South Korea to Los Angeles. He currently takes glargine 20 units at 8 PM every night and rapid acting insulin before meals as part of his basal-bolus regimen. The flight departs 9 AM on February 1st from Seoul and has a 3 hour layover in China. Because the flight crosses the IDL, it arrives the same day at 10 AM in Los Angeles, even though the total travel time is 17 hours. This sounds very confusing, but if you fill out the handout for the patient it turns out to be quite simple.

**Figure 5:**
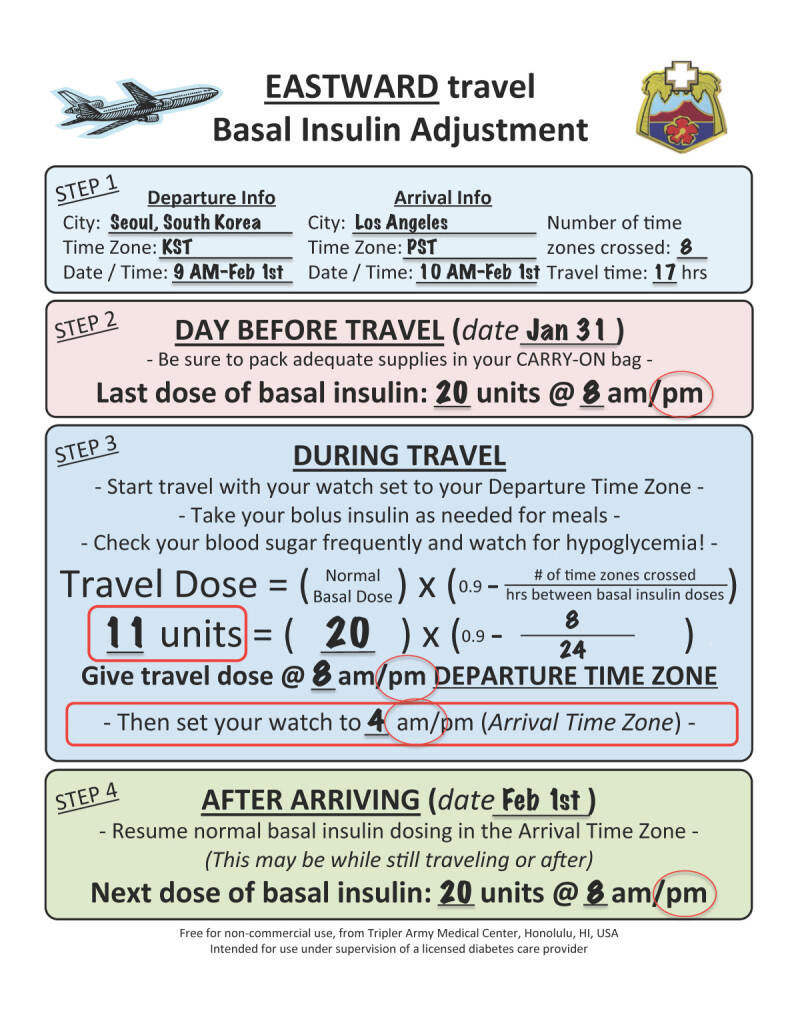
**Calculation for insulin dose change.** This example shows the basal insulin dose change for example 4, scenario 1, Seoul, South Korea to Los Angeles.

Using the Eastward Travel Handout (Figure 5), information on the local time at departure is written down (9 AM KST) as well as the last time basal insulin was given before departure, glargine 20 Units at 8 PM the night before departure. At 8 PM KST only 11 units of glargine are given per the formula on the handout. **AFTER** the reduced basal insulin dose is given, he changes his watch to Los Angeles time (PST). The next dose of glargine is due at 8 PM PST after he arrives, which turns out to be 16 hours after the last dose. This reduced time between doses is why the patient only took just under ^2^/_3_ of their usual glargine dose on the flight. Note that you can disregard the date, and the formula still works!

### Important considerations

Prevention of hypoglycemia while providing adequate insulin coverage is the challenge of diabetes management while traveling, and the use of a split basal insulin dose when traveling west or a reduced basal insulin dose when traveling east offers a safe, simple solution. While there are several published regimens for adjusting insulin for long-distance travel, our method using a single dose change is safe and simple. If following the handout, patients do not even need to know their destination time zone conversion. They simply adjust their watch once per the instructions on the handout.

Despite the simplicity of these instructions, individual patient care and provider preference remain important considerations. For example, even with the 10-20% dose reduction built in to the eastward travel formula (Figure 4), some providers may not feel comfortable giving a reduced dose of glargine for eastward travel, and may want to decrease the next dose of glargine after completion of travel to mitigate the risk of hypoglycemia. Also, if the eastward travel formula shows a requirement for only a small insulin dose to cover just a few hours of time before the next scheduled full dose of basal insulin, many diabetes care providers would recommend skipping that dose, again to mitigate the risk of hypoglycemia. Daylight savings time, observed in the continental United States and some other countries, is yet another consideration, changing the calculated number of time zones between locations by 1 hour depending on the time of year. This small change however is of limited clinical relevance.

## Conclusion

Patients need easy to understand, straightforward instructions for adjusting basal-bolus insulin regimens during travel. Our handouts [see Additional file 1] address most scenarios in a straightforward manner, providing a safe and simple dosing regimen for most extended travel situations.

Our future goal is to move these handouts into an online dosage calculator or smartphone app. For now, we encourage physicians and diabetes providers to use our freely available, simplified handouts to help patients prevent hypoglycemia while safely enjoying their travels.

## Electronic supplementary material

Additional file 1: **Travel handouts.** Detailed yet simple patient handouts allowing for patient-specific basal insulin dosing adjustment recommendations for long distance travel using only a single dose change. (PDF 119 KB)

## Authors’ original submitted files for images

Below are the links to the authors’ original submitted files for images. Authors’ original file for figure 1 Authors’ original file for figure 2 Authors’ original file for figure 3 Authors’ original file for figure 4 Authors’ original file for figure 5

## Abbreviations

EST: Eastern standard time
HST: Hawaii-Aleutian standard time
IDL: International date line
KST: Korean standard time
PST: Pacific standard time
T1D: Type 1 diabetes.
