# Supplementary material for: Extensive clinical experience: a simple guide to basal insulin adjustments for long-distance travel
Source: J Diabetes Metab Disord. 2013 Dec 20;12:59. doi: 10.1186/2251-6581-12-59 (PMC7962589; doi:10.1186/2251-6581-12-59)
Supplement: Supplementary file 1 — Additional file 1: Travel handouts. Detailed yet simple patient handouts allowing for patient-specific basal insulin dosing adjustment recommendations for long distance travel using only a single dose change. (PDF 119 KB) [file 40200_2013_190_MOESM1_ESM.pdf]

WESTWARD travel
Basal Insulin Adjustment

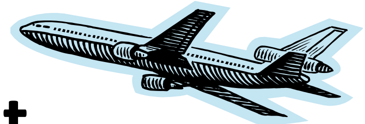

STEP 1

Departure Info

City: ____________________

Time Zone: ______________

Date / Time: _____________

Arrival Info

City: ___________________

Time Zone: _____________

Date / Time: ____________

STEP 2

DAY BEFORE TRAVEL (*date* __________)

- Be sure to pack adequate supplies in your CARRY-ON bag -

Last dose of basal insulin: ___ units @ ____ am/pm

STEP 3

DURING TRAVEL

- Start travel with your watch set to your Departure Time Zone -

- Take your bolus insulin as needed for meals -

- Check your blood sugar frequently and watch for hypoglycemia! -

At ______ am/pm DEPARTURE TIME ZONE

take ½ of your "usual" basal insulin dose = ___ units

- Then set your watch to _____ am/pm (*Arrival Time Zone*) -

At ______ am/pm ARRIVAL TIME ZONE

take ½ of your "usual" basal insulin dose = ____ units

(This may be while still traveling or after arrival depending on the time)

STEP 4

AFTER ARRIVING (*date* __________)

- Resume normal basal insulin dosing in the Arrival Time Zone -

Next dose of basal insulin: ___ units @ ____ am/pm

Free for non-commercial use, from Tripler Army Medical Center, Honolulu, HI, USA
Intended for use under supervision of a licensed diabetes care provider

From ***Pinsker et al.***: **Extensive clinical experience: a simple guide to basal insulin adjustments for long-distance travel.** ***Journal of Diabetes & Metabolic Disorders*** 2013 **12**:59.

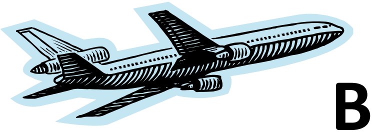

EASTWARD travel Basal Insulin Adjustment

STEP 1

**Departure Info**	**Arrival Info**	
City: ________________	City: ________________	Number of time
Time Zone: ___________	Time Zone: ___________	zones crossed: _____
Date / Time: __________	Date / Time: __________	Travel time: _____ hrs

STEP 2

DAY BEFORE TRAVEL (*date* ________)

- Be sure to pack adequate supplies in your CARRY-ON bag -

Last dose of basal insulin: ___ units @ ____ am/pm

STEP 3

DURING TRAVEL

- Start travel with your watch set to your Departure Time Zone -

- Take your bolus insulin as needed for meals -

- Check your blood sugar frequently and watch for hypoglycemia! -

$$\text{Travel Dose} = \left(\text{Normal Basal Dose}\right) \times \left(0.9 - \frac{\text{\# of time zones crossed}}{\text{hrs between basal insulin doses}}\right)$$

$$\text{\_\_\_\_\_ units} = \left(\quad\quad\right) \times \left(0.9 - \frac{\quad}{\quad}\right)$$

Give travel dose @ ____ am/pm DEPARTURE TIME ZONE

- Then set your watch to _____ am/pm (*Arrival Time Zone*) -

STEP 4

AFTER ARRIVING (*date* ________)

- Resume normal basal insulin dosing in the Arrival Time Zone -

(This may be while still traveling or after)

Next dose of basal insulin: ___ units @ ____ am/pm

Free for non-commercial use, from Tripler Army Medical Center, Honolulu, HI, USA
Intended for use under supervision of a licensed diabetes care provider

From ***Pinsker et al.***: **Extensive clinical experience: a simple guide to basal insulin adjustments for long-distance travel.** ***Journal of Diabetes & Metabolic Disorders*** 2013 **12**:59.
