# Supplementary figures and images for: Extensive clinical experience: a simple guide to basal insulin adjustments for long-distance travel
Source: J Diabetes Metab Disord. 2013 Dec 20;12:59. doi: 10.1186/2251-6581-12-59 (PMC7962589; doi:10.1186/2251-6581-12-59)

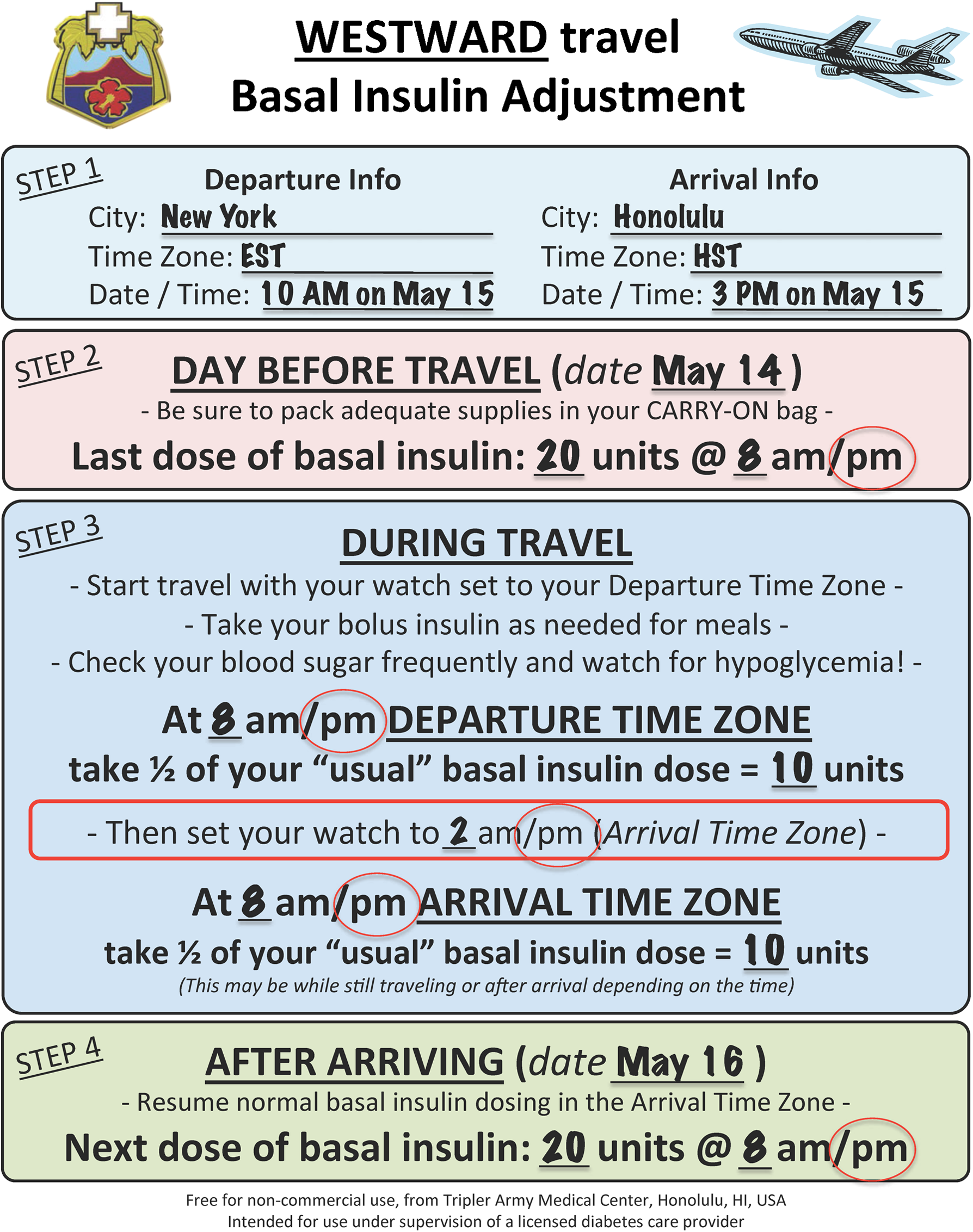

Supplement: Supplementary file 2 — Authors’ original file for figure 1 [file 40200_2013_190_MOESM2_ESM.tif]

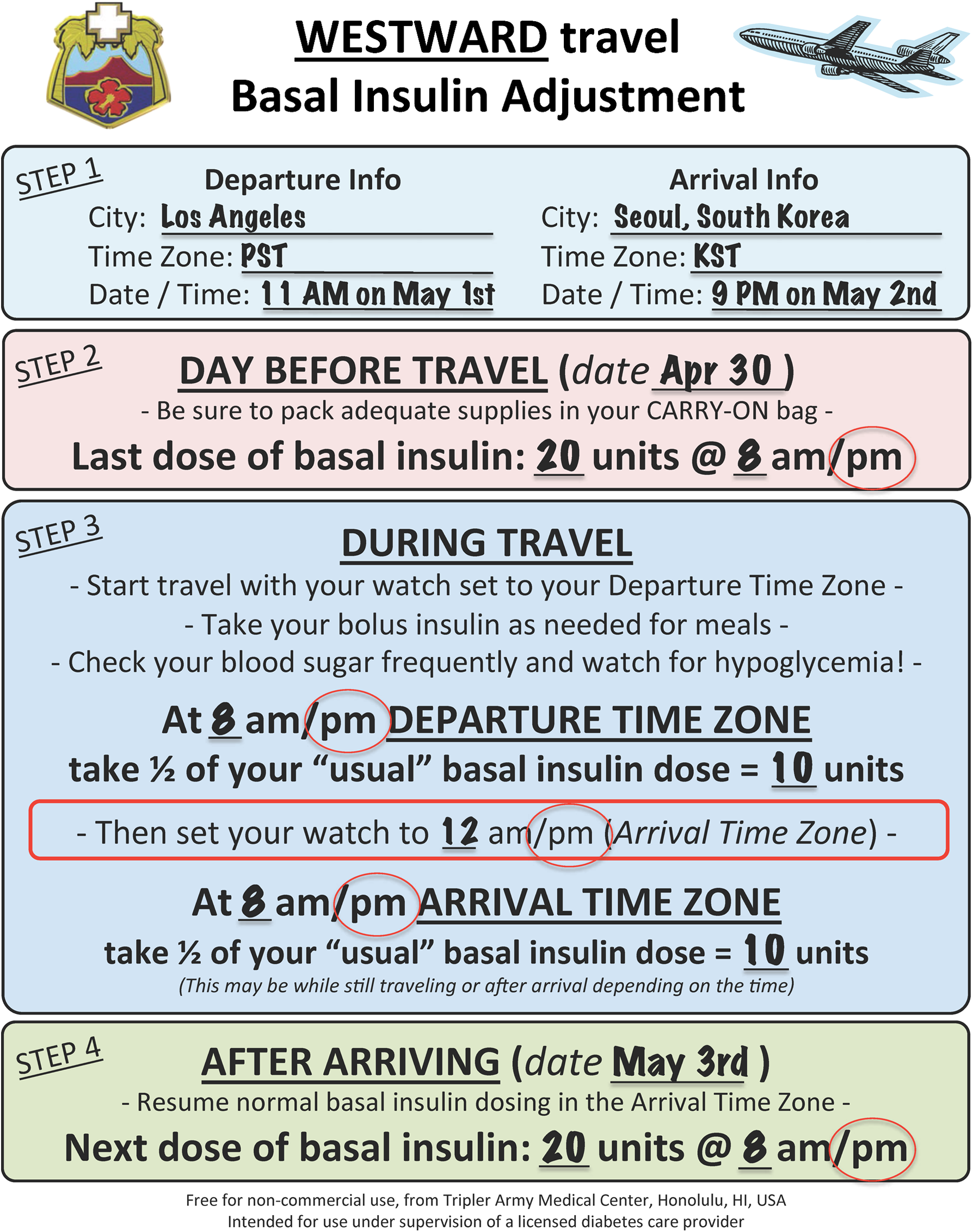

Supplement: Supplementary file 3 — Authors’ original file for figure 2 [file 40200_2013_190_MOESM3_ESM.tif]

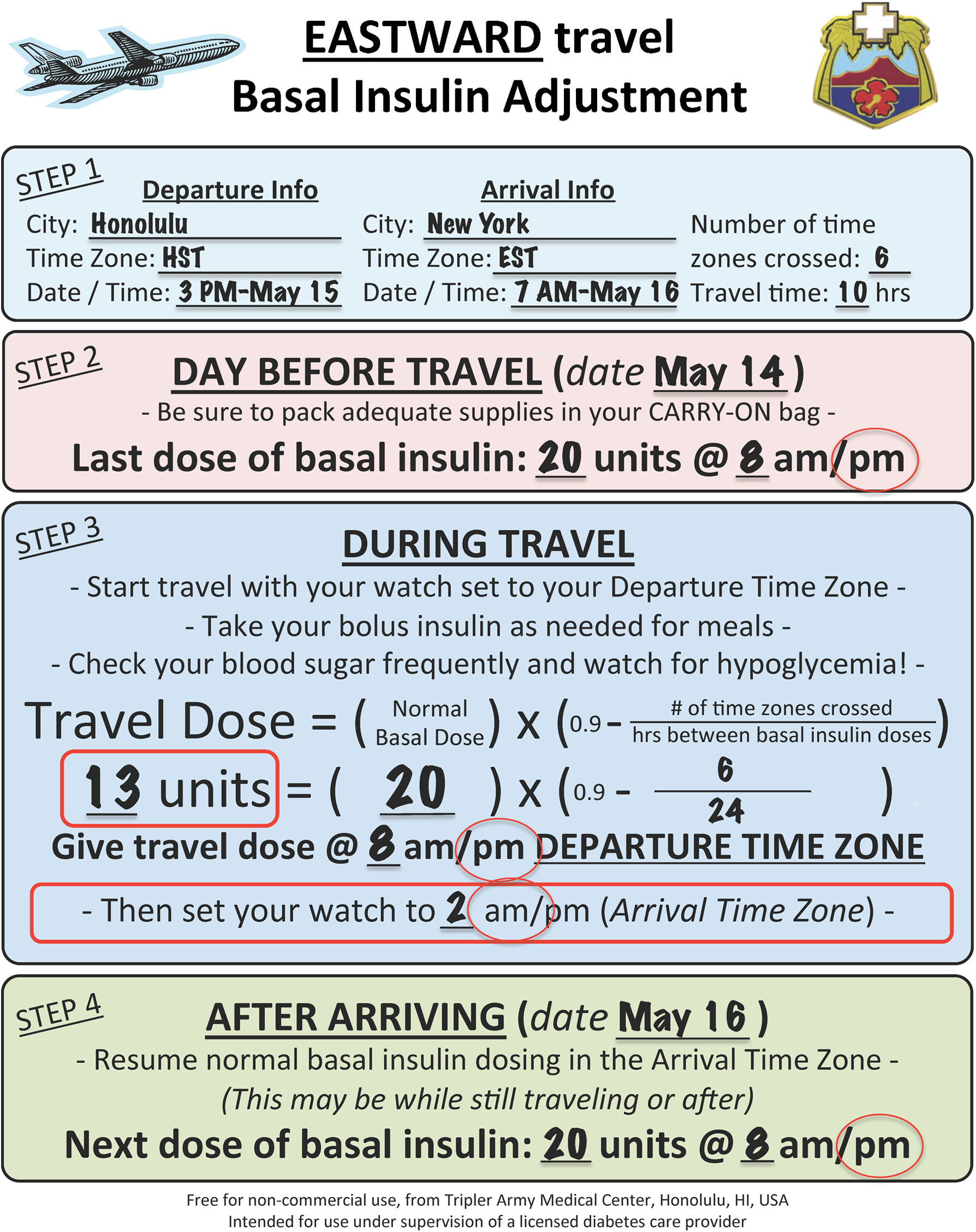

Supplement: Supplementary file 4 — Authors’ original file for figure 3 [file 40200_2013_190_MOESM4_ESM.tif]

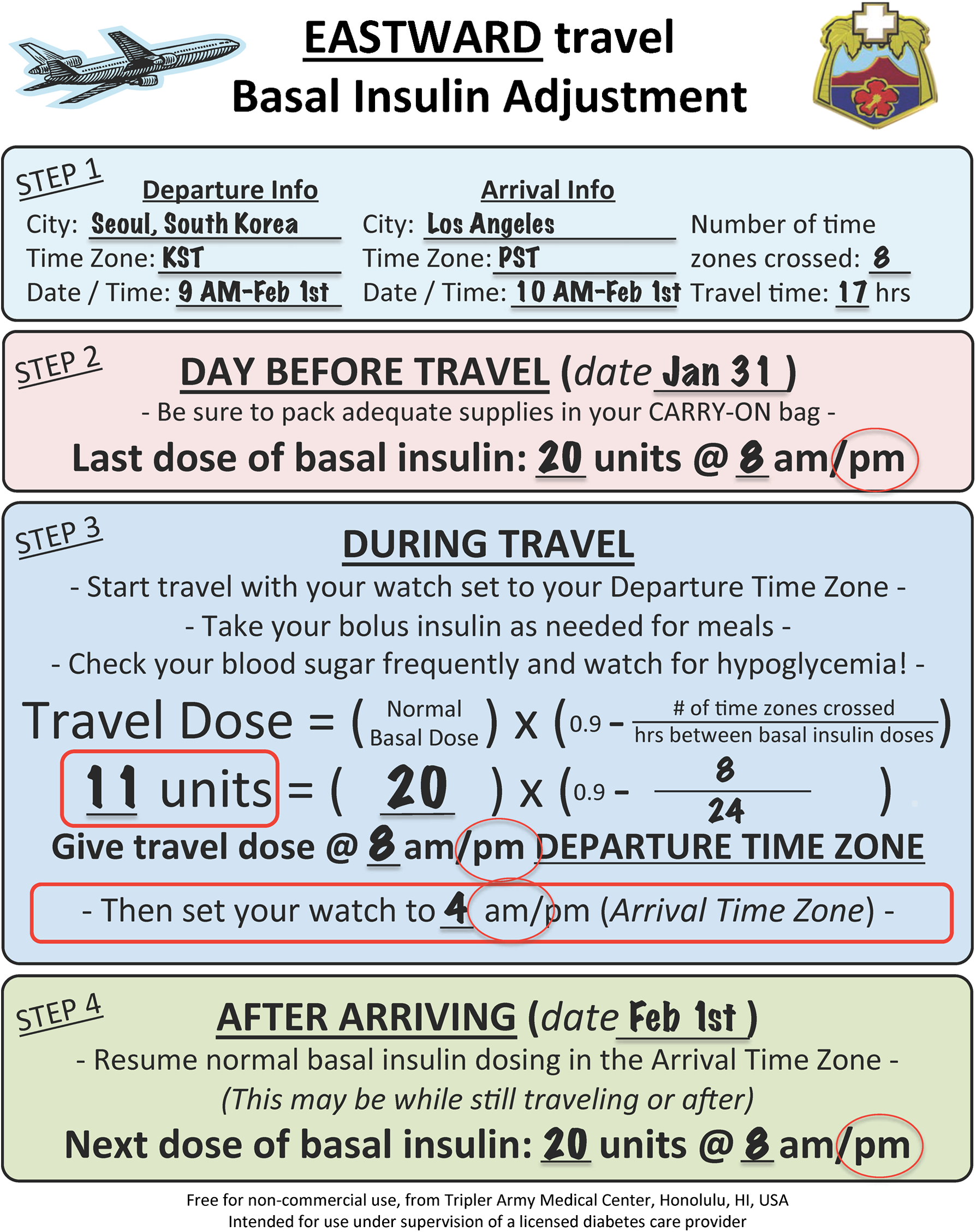

Supplement: Supplementary file 6 — Authors’ original file for figure 5 [file 40200_2013_190_MOESM6_ESM.tif]
