# Supplementary material for: Extensive clinical experience: a simple guide to basal insulin adjustments for long-distance travel
Source: J Diabetes Metab Disord. 2013 Dec 20;12:59. doi: 10.1186/2251-6581-12-59 (PMC7962589; doi:10.1186/2251-6581-12-59)
Supplement: Supplementary file 5 — Authors’ original file for figure 4 [file 40200_2013_190_MOESM5_ESM.pdf]

$$Travel\ Dose = Normal\ Dose \times 0.9 - \frac{\#\ of\ Time\ Zones\ Crossed}{Hours\ Between\ Basal\ Insulin\ Doses}$$

To calculate the new dose to use on the handout, use the formula and enter:

1) The normal basal insulin dose.
2) The number of time zones crossed. Note this may be different than the time difference between the locations if crossing the IDL. For example, Seoul, South Korea is 8 time zones away from Los Angeles, even though they have a 16 hour time difference. Use of a time zone map (www.worldtimezone.com/wtz-pacific24.php) can help to determine how many time zones are traversed.
3) Hours between basal insulin doses. Enter 24 if giving insulin once daily. For twice daily basal insulin, it would be 12 hours.
4) As noted in the *Important Considerations* section, the travel dose offered by the formula is a recommendation, and providers may want to adjust the dose for each individual patient.

* Adapted and modified from the Saskatchewan Advanced Insulin Dose Adjustment Module [9].
